# Comparison of anxiety, pain, and quality of life in individuals with mild or moderate malocclusion between conventional fixed orthodontic treatment versus Invisalign: a randomised clinical trial

**DOI:** 10.1186/s12903-024-04335-1

**Published:** 2024-05-17

**Authors:** Yasemin Tunca, Yesim Kaya, Murat Tunca, Sıddık Keskin

**Affiliations:** 1 Faculty of Dentistry, Department of Orthodontics, Van Yuzuncu Yil University, Van, 65080 Turkey; 2 Faculty of Dentistry, Department of Orthodontics, Ankara Yıldırım Beyazıt University, Ankara, Turkey; 3 Faculty of Medicine, Department of Biostatistics, Van Yuzuncu Yil University, Van, 65080 Turkey

**Keywords:** Clear aligner, Anxiety, Pain, Quality of Life

## Abstract

**Background:**

We evaluated anxiety, pain, and oral-health-related quality of life in individuals treated with conventional fixed appliances (Group A) and clear aligners (Group B) for moderate malocclusion during the initial phase of orthodontic treatment.

**Methods:**

Sixty individuals, separated into Group A (*n* = 30) and Group B (*n* = 30), were included in the study. They completed the Anxiety Levels, Oral Health Impact Profile-14, and Oral Health Related Quality of Life - United Kingdom/Surveys after the application of attachments on days 0 (T1), 10 (T10), and 20 (T20). Their pain levels were evaluated with the Visual Analogue Scale on days 0, 2, and 6 in the 2nd and 6th hours and on the 1st, 3rd, 7th, 14th, and 21st days.

**Results:**

Per the VAS questionnaire, pain levels in the 2nd hour, 6th hour, 1st day, and 3rd day were significantly lower in Group B than in Group A. In the OHIP-14 survey results, the comparison between Group A and Group B showed a significant difference only on the 1st day. The STAI and OHRQoL-UK survey results did not differ significantly between the groups.

**Conclusions:**

We found no significant difference between the two groups in terms of anxiety levels, and pain among individuals in Group A was higher than in Group B only at the beginning of the treatment. No significant differences were observed in terms of individuals’ quality of life.

**Trial registration:**

NCT06133296 (retrospectively registered)- Registration Date:15/11/2023.

## Background

Clear-aligner treatment is an increasingly popular orthodontic treatment option [1]. With this treatment method, which was introduced in the literature at the beginning of the 20th century, approximately four million individuals in 2019 and over twelve million individuals today have been treated [2]. In addition, the number of commercial companies worldwide producing clear aligners has reached twenty-seven, indicating that these figures will increase rapidly [2, 3]. Although there are debates about the treatment’s effectiveness, the increase in living standards and the improvement of the quality of life of individuals relative to conventional fixed orthodontic treatment have increased interest in clear aligner treatment [4–9].

The World Health Organization has multidimensional definitions of the concepts of “quality of life” and “health.” Their commonality is that they emphasize the importance of individuals’ psychological and social status in recent years [10–12]. Quality of life is affected by dentofacial problems caused by malocclusions as well as the psychosocial state of individuals during orthodontic treatment [11, 13]. Oral-health-related quality of life was defined as “the absence of physical and psychological problems in terms of oral health and self-confidence associated with the maxillofacial region,” and the importance of self-confidence and psychosocial status that could affect quality of life was emphasized [14]. The pain and anxiety experienced before and during orthodontic treatment are among the factors that influence oral health-related quality of life [15].

Pain is an emotional state individuals undergoing orthodontic treatment frequently encounter, leading to cooperation problems and even causing patients to give up treatment [16]. In studies evaluating pain levels in conventional fixed orthodontic treatments, with the first arch wire application, pain peaks at the 4th hour of the 1st day and can be felt until the 7th day [6, 17, 18]. When comparing pain levels between individuals treated with conventional fixed orthodontic appliances and clear aligners, clear aligner treatment was found to be less painful in the first few days, but there was no significant difference in pain levels at later stages of treatment [19, 20].

The importance of malocclusion type and arch length discrepancy has been emphasized in studies comparing conventional fixed orthodontic treatment and clear aligner treatment in terms of pain and quality of life [19, 20]. Considering that the concepts of anxiety, pain, and quality of life are interrelated, there are limited studies in which the two treatment methods are evaluated in terms of these factors in a particular malocclusion [21]. We aim to compare anxiety and pain values observed in the initial phase of orthodontic treatment and oral-health-related quality of life among individuals with a moderate malocclusion treated with conventional fixed orthodontic appliances and clear aligners. Our null hypothesis (H0) is that there is no difference in anxiety, pain, or oral-health-related quality of life between individuals treated with conventional fixed orthodontic appliances and clear aligners.

## Methods

### Trial design

This study is a single-centre two-arm parallel-group randomized clinical trial with a 1:1 allocation ratio. This research was carried out between 01/03/2021-01/03/2023. The XXX University Non-Interventional Clinical Research Committee approved the study (2021/02/08; Clinical trial registration number: NCT06133296 retrospectively registered- Registration Date:15/11/2023). This randomized clinical trial study included sixty individuals who underwent conventional fixed orthodontic treatment/and aligner treatment at XXXX University Faculty of Dentistry, Department of Orthodontics. Written informed consent was obtained from all individuals who agreed to participate in our study, which was conducted under the guidance of the Helsinki Declaration ethical rules and from the parents of patients younger than 18 years of age after they received detailed information about the purpose and method of the study.

### Inclusion and exclusion criteria

The study included individuals who had not undergone orthodontic treatment, had Angle Class I malocclusion, had a 4–6 mm arch length discrepancy in both dental arches, were in the permanent dentition period, had missing or impacted teeth, had plaque accumulation, had gingival inflammation, were non-smokers, and were non-alcohol drinkers. The study excluded individuals who had undergone extraction fixed orthodontic treatment, experienced radiologically observed alveolar bone loss, had any systemic disease, or reported using drugs or analgesics during the survey.

### Establishment of working groups

Group A (*n* = 30) comprised individuals who underwent conventional fixed orthodontic treatment and group B (*n* = 30) individuals who underwent clear aligner treatment. Individuals were randomized, using closed envelopes, into the two groups. Individuals with fixed orthodontic attachments attached to all their permanent teeth and individuals with planned tooth movement for 80% of their permanent teeth were included in Group B. Digital orthodontic models were created with a three-dimensional model scanning device (iTero Element 2, Align Technology, San Jose, CA). Arch length discrepancy (discrepancy Index) was calculated using the OrthoCAD (Align Technology, Inc.) program in digital orthodontic models transferred to a computer environment. Note that the groups were similar in terms of age and gender. Bonding and surveys were conducted in the morning to standardize the values to be measured for all three surveys. Before the application of fixed orthodontic attachments in Group A and clear aligners in Group B, one researcher recorded the participants’ age, gender, arch length deviation, and education level and the bonding of the attachments in both groups in the study, (.) and another researcher recorded the survey results (.).

### Bonding of fixed orthodontic attachments

Roth brackets (0.018; Gemini Roth System, 3 M Unitek, USA) were used for the fixed orthodontic attachment. Tooth surfaces were etched with 35% gel phosphoric acid (ScotchbondTM Universal Etchant, 3 M Unitek, Monrovia, CA, USA) for 30 s and then washed with water for 15 s. A primer (Transbond XT, 3 M Unitek, USA) was applied to the etching surface with the manufacturer’s applicator. After cleaning the adhesive residues protruding from the edges of the bracket base with the help of a probe, each tooth was irradiated with LED light device (D-Light Pro, GC Corporation, Leuven, Belgium for 20 s for 5 s from the mesial, distal occlusal and gingival surfaces. For leveling after bonding, 0.012 nickel titanium arch wires (3 M Unitek Monrovia, CA, USA) were tied with an elastic ligature.

### Application of clear aligners

For Group B, clear aligners were ordered after the recordings were evaluated in the Clincheck program, and the final treatment plan was created. After the clear aligners came from the manufacturer (Align Technology, Santa Clara, CA), the guide aligner’s compatibility with each patient’s mouth was checked for attachments. The enamel surfaces on which the attachments will be applied were etched with the same method as in Group A. After an adhesive (Reliance Orthodontic Products Inc., Itasca, IL, USA) was condensed into the attachment spaces inside the guide plate, the guide aligner was placed in the correct position in the mouth and each attachment was applied on the buccal surface with an LED light device (D-Light Pro, GC Corporation, Leuven, Belgium) for 20 s. After the guide aligner was removed, the composite residues around the attachments were cleaned and the first treatment aligner was applied. The individuals were informed that they should use their clear aligner continuously, except during meals, and replace them after 10 days.

### Collection of data

To measure the individuals’ anxiety level, we applied the Spielberger State and Trait Anxiety Inventory (STAI) survey. It includes 40 questions that measure state anxiety (STAI-S, 20) and trait anxiety (STAI-T, 20). The answer categories for the questions varied according to the nature of the problem (e.g., 1: No, 2: slightly, 3: multiple, and 4: always) in the form of a four-point scale. There are two types of expressions on the scales: direct and reversed. Direct expressions indicate negative emotions, and reversed expressions indicate positive emotions. The reversed expressions were scored as follows: those that were worth 1 point were converted to 4 points, and those that were worth 4 points were converted to 1 point. Answers of 4 in direct expressions and answers of 1 in reversed expressions show high anxiety. Both groups were applied to both groups before treatment (T0), fixed orthodontic attachments in group A and after the first clear aligner was applied in group B (T1), on the 10th day (T10), and on the 20th day (T20).

A 10 cm-long scale called the Visual Analogue Scale (VAS) was used to measure the individuals’ pain levels. On the scale, 0 denotes painlessness and 10 denotes unbearable pain. Evaluations were conducted at 0, 2, and 6 h after treatment and on days 1, 3, 7, 14, and 21.

The Oral Health Impact Profile-14 (OHIP-14) and Oral-Health-Related Quality of Life-United Kingdom (OHRQoL-UK) questionnaires were used to measure quality of life in our study. The OHIP-14 questionnaire consists of seven domains and 14 questions, including those related to functional limitation, physical pain, psychological discomfort, physical disability, psychological disability, and disability. The individuals were asked to answer each question as follows: 0: never, 1: very little, 2: sometimes, 3: quite often, and 4: often. After the results were collected separately for each of the seven subgroups, they were added together to calculate the total score. High scores on the OHIP-14 questionnaire indicate that quality of life was adversely affected. The OHRQoL-UK questionnaire comprises 16 questions in four categories: symptom (two questions), physical condition (five questions), psychological condition (five questions), and social situation (four questions). The participants responded on a Likert scale, with 1 point for very bad influence, 2 for bad influence, 3 for no effect, 4 for good effect, and 5 for very good effect. Possible total scores range from 16 to 80. A high score indicates a good quality of life for oral and dental health whereas a low score indicates a poor quality of life for oral and dental health. In both groups, after fixed orthodontic attachments in Group A and after the first clear aligner was applied in Group B (T1) and after 10 days (T10) and 20 days (T20) of treatment, the OHIP-14 and OHRQoL-UK questionnaires were administered.

### Sample size calculation

The sample size was calculated using the G-Power statistical package software (Version 3.1 Franz Foul, Universität Kiel, Germany). When we calculated the sample for the effect size (d, effect size = 0.8), type I error (α = 0.05), and 80% power values, we calculated a sample size of 52 individuals for the two independent groups [22]. However, it was deemed appropriate to include a total of 60 individuals, assuming that the rate of exclusion from the study would be 15%. Since the study was completed with 60 individuals, the post hoc power was determined as 88%.

### Statistical analysis

Descriptive statistics were presented as means and standard deviations for continuous variables and as counts and percentages for categorical variables. Two sample t tests were conducted to compare independent two groups for age and arch length disperancy. In addition, a two-way repeated measure ANOVA was conducted to determine whether there is a difference between groups (independent) and periods (dependent) in terms of other characteristics. To determine linear relationships between the continuous variables, the Pearson correlation coefficient was computed. A Chi-square test was conducted to determine the relationship between categorical variables. Statistical significance level was set st 5%, and the SPSS for Windows version 21.0 (SPSS Inc., Chicago, Illionois, USA) statistical package program was used for all statistical computations.

## Results

Table 1 presents the descriptive statistics for the age, gender, arch length deviation, and educational level of the individuals who were included in the study. The average age of the individuals was 21.3 ± 3.37 years in Group A and 23.65 ± 6.58 years in Group B, and the difference between the groups was not statistically significant (*p* = 0.612). The arch length deviation was 5.01 ± 0.55 mm in Group A and 4.94 ± 0.66 mm in Group B, and the difference was not statistically significant (*p* = 0.656).

**Table 1:** Demographic data of group A and group B

		Group A	Group B	*p*
Age (year)	Mean ± SD	21.3 ± 3.37	23.65 ± 6.58	*0.612* ^#^
	Min	17.08	17.41	
	Max	24.9	28	
Gender	Female	15 (%50)	15 (%50)	0.999^φ^
	Male	15 (%50)	15 (%50)	
Arch Length Disperancy	Mean ± SD	5.01 ± 0.55	4.94 ± 0.66	*0.656* ^#^
	Min	4.1	4.0	
	Max	5.9	5.9	
Educational Level	High School	14 (%46.7)	2 (%6.7)	
	Undergraduate	15 (%50)	13 (%43.3)	
	Graduate	1 (%3.3)	15 (%50)	

^#^ : Independent two sample t test.

^φ^ : Chi-Square test.

**Fig. 1:**
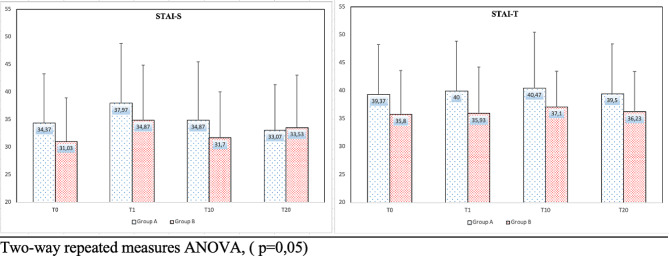
Comparison of anxiety level (STAI-S, STAI-T) between the the group A and the group B

Figure 1 presents the results of the STAI-T and STAI-S surveys of the individuals who participated in the research. The STAI-S values were 34.37 ± 8.94 in T0, 37.97 ± 10.85 in T1, 34.87 ± 1056 in T10, and 33.07 ± 8.28 in T20 in Group A and 0.03 ± 7.90 in T0, 34.87 ± 9.95 in T1, 31.70 ± 8.33 in T10, and 33.53 ± 9.53 in T20 in Group B. The STAI-T values were 39.37 ± 8.86 in T0, 40.00 ± 8.90 in T1, 40.47 ± 10.03 in T10, and 39.50 ± 8.93 in T20 in Group A and 35.80 ± 7.84 in T0, 35.93 ± 8.29 in T1, 37.10 ± 6.38 in T10, and 36.23 ± 7.21 in T20 in Group B. In terms of STAI-S and STAI-T values, the changes observed within and between the groups were not significant.

**Fig. 2:**
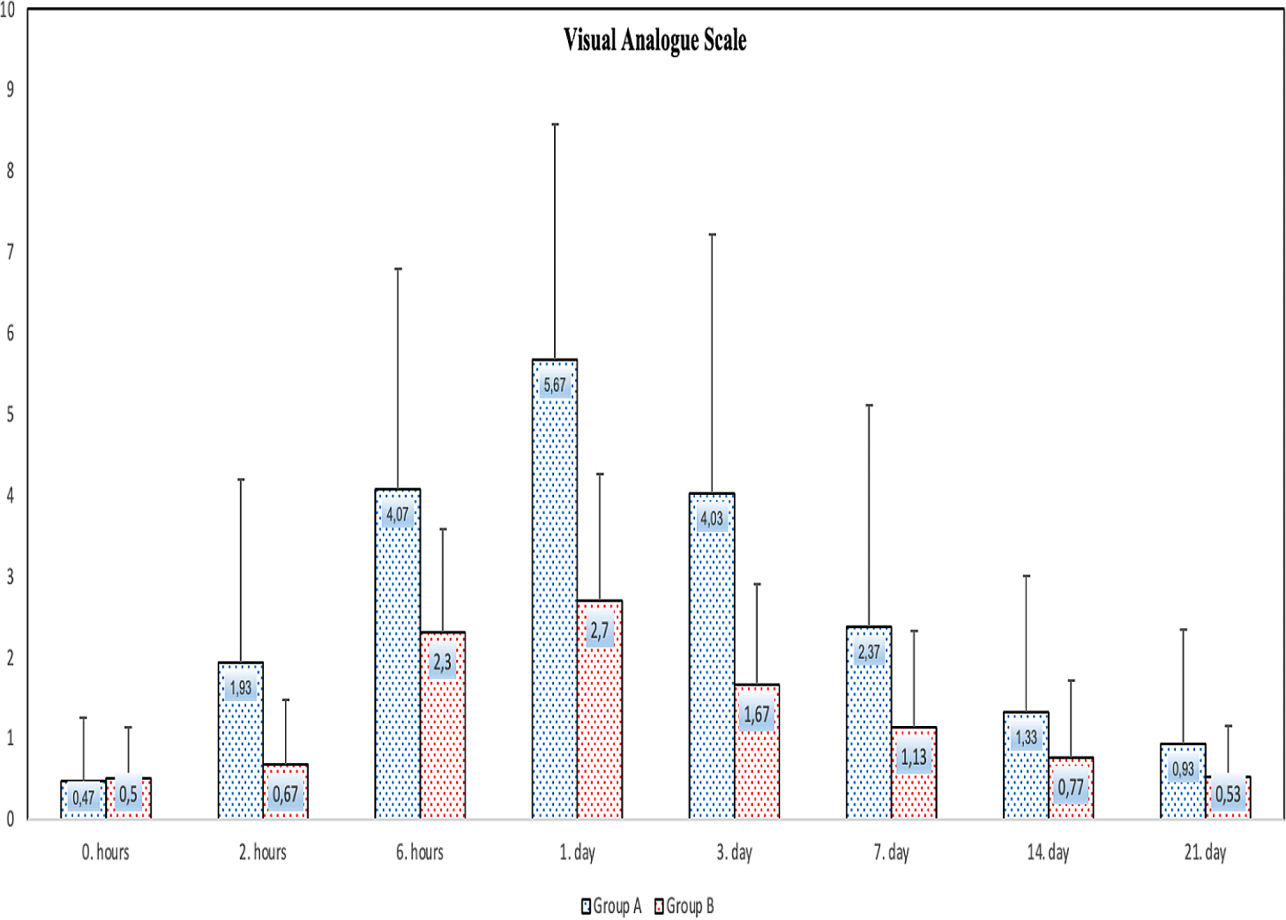
Comparison of VAS pain level between the the group A and the group B

Figure 2 shows a comparison of pain levels between Group C and Group A. The median (maximum − minimum) values of pain levels at 0, 2, and 6 h and on the 1st, 3rd, 7th, 14th, and 21st days were 0 (0 − 3), 1.5 (0 − 10), 4 (0 − 10), 5.5 (0 − 10), 3 (0 − 10), 1.5 (0 − 10), 1 (0 − 5), and 0 (0 − 6) in Group B; and 0 (0 − 2), 0.5 (0 − 3), 2 (0 − 5), 3 (0 − 6), 1.5 (0 − 4), 1(0 − 4), 0.5 (0 − 3), and 0 (0 − 2) in Group A. At the 2nd hour (*p* = 0.004), 6th hour (*p* = 0.008), 1st day (*p* = 0.001), and 3rd day (*p* = 0.004), pain levels were found to be significantly lower in Group B compared to those in Group A.

**Fig. 3:**
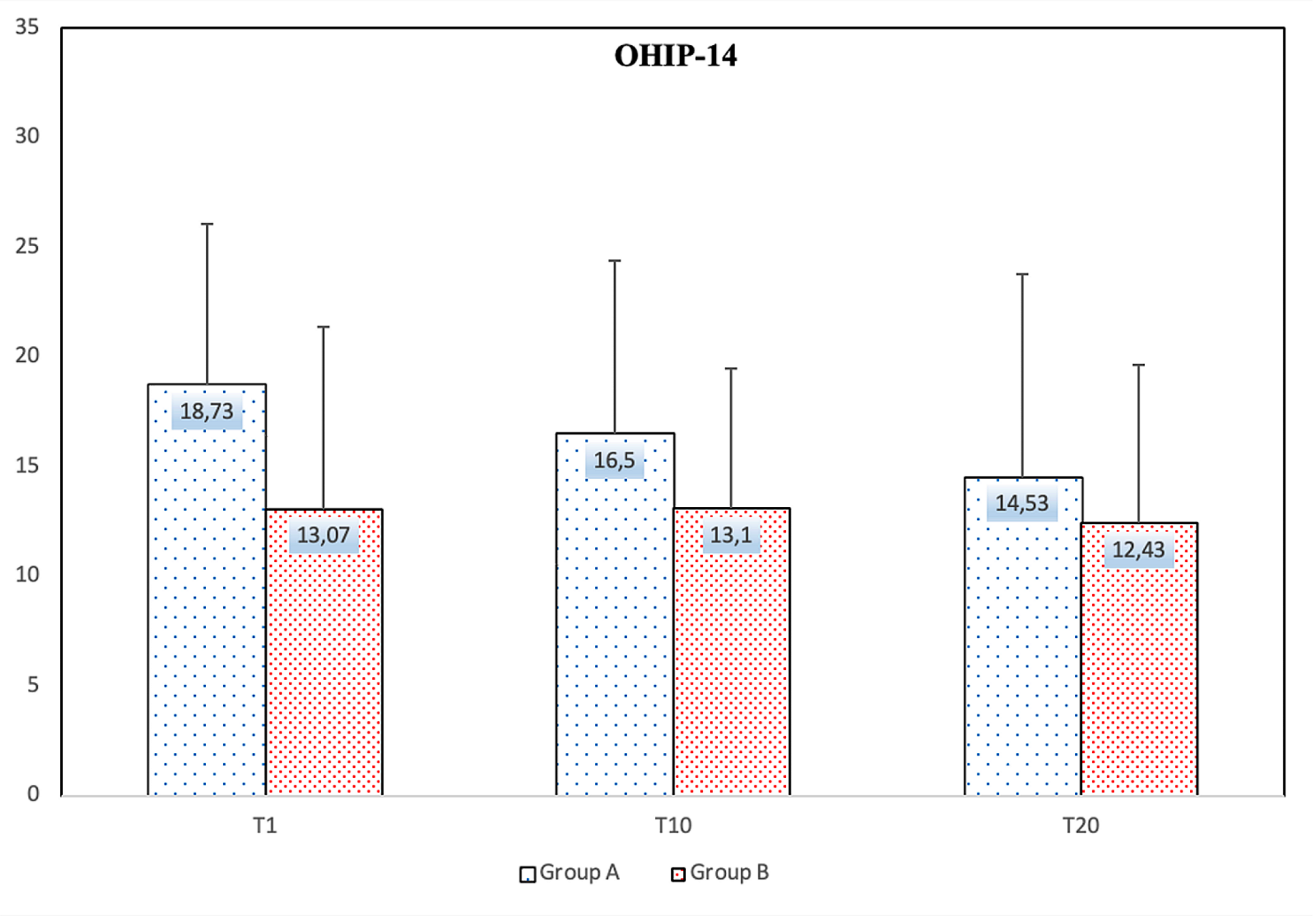
Comparison of OHIP-14 between the group A and the group B

**Fig. 4:**
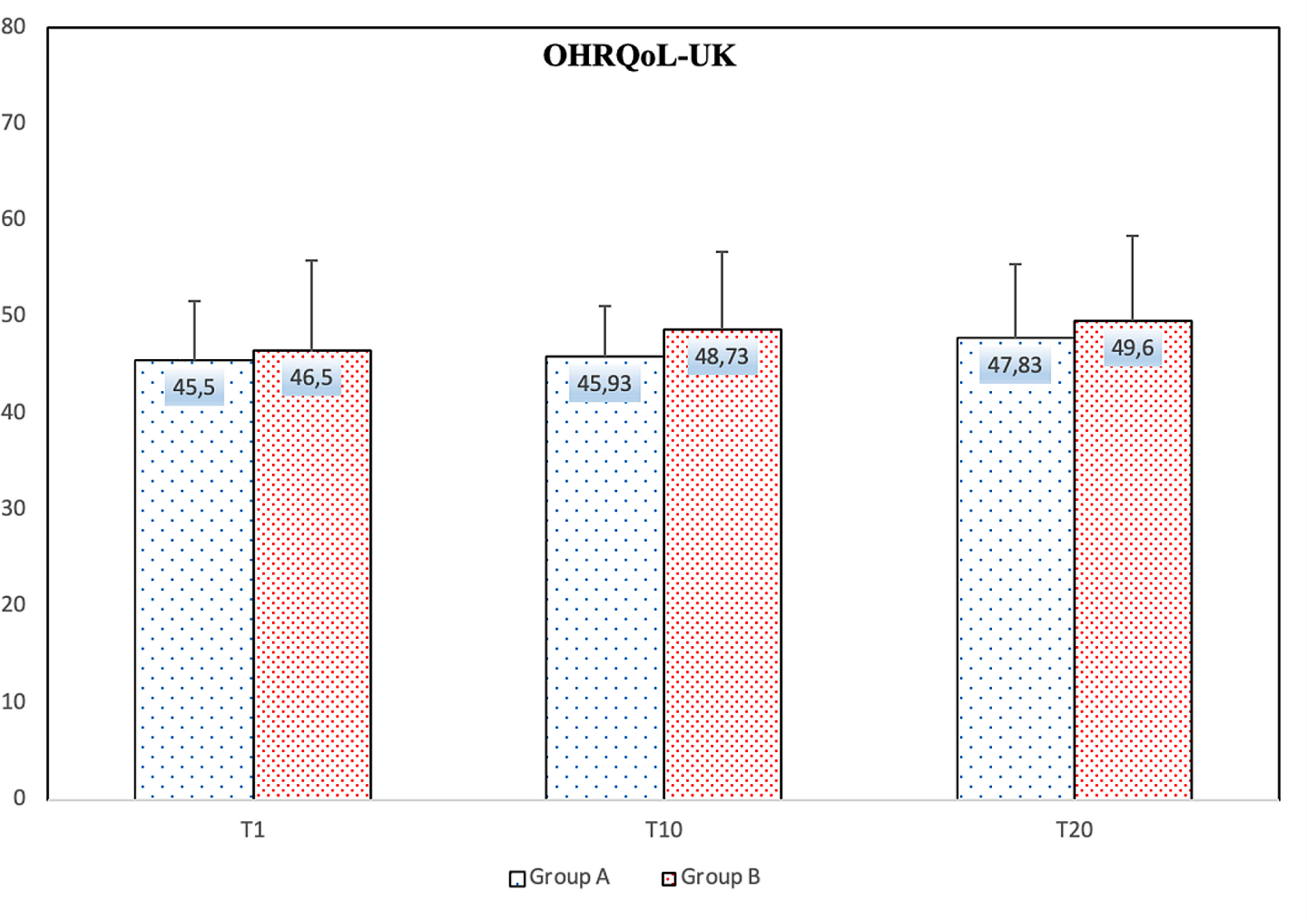
Comparison of OHRQOL-UK between the group A and the group B

Figure 3 shows the OHIP-14 survey results of the research participants, while Fig. 4 shows the results of the OHRQoL-UK survey. The OHIP-14 survey results on days 1, 10, and 20 were 18.73 ± 7.75, 16.50 ± 7.45, and 14.53 ± 7.07 in Group A and 13.07 ± 7.32, 13.10 ± 7.91, and 12.43 ± 9.24 in Group B, respectively. The comparison results between the groups showed a significant difference only on the 1st day (*p* = 0.049). The OHRQoL-UK survey results on days 1, 10, and 20 were 45.50 ± 6.16, 45.93 ± 5.29, and 47.83 ± 7.65 in Group A and 46.50 ± 9.34, 48.73 ± 8.02, and 49.60 ± 8.87 in Group B, respectively. No significant difference was observed between the groups.

## Discussion

Although the effectiveness of the treatment is debated, studies evaluating the pain, anxiety and quality of life associated with clear aligners that have become popular in the last two decades have shown conflicting results [4, 6, 23, 24]. In the present study, pain levels were significantly lower in Group B at the 2nd and 6th hours and on the 1st and 3rd days. According to the OHIP-14 survey results, only during the 1st day was the quality of life significantly better in Group A, and there was no significant difference between the groups, according to the OHRQoL-UK survey results. In light of these results, the null hypothesis of our research was partially supported.

Dental anxiety varies between 10% and 29.3% [25]. In two studies evaluating the anxiety levels of individuals treated with the straight-wire technique using conventional metal brackets in the first month, the anxiety level increased significantly after the first arch wire placement and gradually decreased within 30 days. Age and gender did not have a significant effect on anxiety [13, 26]. Sarı et al. [27] compared the anxiety levels of individuals who will receive orthodontic treatment, individuals who received orthodontic treatment, and the parents of both groups and stated that the anxiety levels of individuals to be treated and their families were higher. They stated that environmental factors, such as family, should be considered in the evaluation of anxiety levels. In our study, the STAI questionnaire Spilberger [28] developed, which is also used in current studies, was used to evaluate individuals’ state and trait anxiety levels.

Gao et al. [29] compared conventional fixed orthodontic and Invisalign™ treatments in terms of anxiety levels. The anxiety level was significantly lower in the group treated with Invisalign™. In addition, anxiety levels increased on the first day in both groups and then gradually decreased until the 14th day. In our study, we observed that STAI-S values increased significantly in both groups after treatment began. However, there was no significant difference between the time periods in terms of the STAI-S and STAI-T values. The differences in the results may have arisen due to the inclusion of individuals with heterogeneous malocclusions in the study [29].

In a conventional fixed orthodontic treatment, immediately after the initial arch wire application, edema and acute ischemia in periodontal tissues are seen together with pain [30]. Approximately 95% of individuals feel pain during conventional fixed orthodontic treatment, and 8% stop treatment because of pain [18, 31]. The pain begins within 12 h after arch wire application, peaks within one day, and gradually decreases after the 3rd day [30, 32]. In studies comparing pain levels between individuals treated with fixed orthodontic treatment and clear aligner treatment, Fujimaya et al. [33] found that pain levels peaked in the first 24 h in both groups and then gradually decreased. In addition, no pain was observed after the 5th day in either group, and although it was significant only on the 3rd and 4th days, the pain level in the first four days was lower in the clear aligner group. Gao et al. [29] reported that pain peaked in the first 24 h and was significantly lower in the clear aligner group during the 14-day evaluation period. Similarly, other studies have shown that individuals treated with clear aligners feel less pain than individuals treated with conventional fixed orthodontic appliances [8, 34, 35].

In contrast, Cardoso et al. [19] and Mheissen et al. [20] stated in their systematic reviews that the pain level was lower, especially in individuals who underwent clear aligner treatment at the beginning of the treatment. They also emphasized that the studies they evaluated presented low or moderate evidence that the sensation of pain could vary between individuals and that the severity of malocclusion was affected the level of pain [19, 20]. Yassir et al. [34] reported that clear aligners were more effective in correcting mild to moderate malocclusions. To minimize the factors that may affect pain level, we included individuals with angle class I malocclusion and 4 − 6-mm arch length deviation in both dental arches in our study. However, attention was paid to planning tooth movement for 80% of the permanent teeth in the clear-aligner group. As a result, pain in Group A was significantly lower in the 2nd and 6th hours and on the 1st and 3rd days, in line with the findings of the current studies.

Antonio-Zancajo et al. [21] compared conventional fixed orthodontic and clear aligner treatments in terms of quality of life and stated that it was significantly higher in individuals treated with clear aligners. Alfawal et al. [35] used the OHIP-14 survey and found that individuals treated with clear aligners had higher quality of life scores and shorter treatment periods, but the type of malocclusion may have affected these results. Sauer et al. [36] reported that clear aligners slightly enhanced patients’ quality of life. Sharma et al. [37] used the COHIP-SF 19 survey and found no significant difference between the two groups in terms of quality of life. Given that many factors affect quality of life, the results of our study, which used two different surveys, were compatible with those of Sharma et al. [37]

Studies evaluating quality of life have shown differences in results [8, 9, 19, 20, 29, 33, 38]. Malocclusion types and crowding levels should be considered in studies evaluating the relationship between orthodontic pain and quality of life [19, 20]. Therefore, we aimed to evaluate individuals with a specific malocclusion group and arch length discrepancy. In addition, the biomechanics of conventional fixed orthodontic and clear-aligner treatments are different, and the attachments used in conventional fixed orthodontic treatment may cause soft tissue disorders, which may also have caused the differences in the results. In addition, considering the previous studies, most of which were short-term, we think that movement starts immediately in all teeth in conventional fixed orthodontic treatment, and that the number of clear aligners, the duration of use, attachment types, and the order of tooth movement planning affect the results in individuals receiving clear aligner treatment.

The treatment satisfaction of individuals treated with clear aligners and their psychological well-being, eating and drinking comfort, participation in activities in social life, and aesthetics, and clear aligners can be effective in the selection of this treatment method [36, 37, 39, 40]. Both treatment methods can reduce patient comfort due to soft tissue incompatibility with fixed orthodontic attachments and clear aligners as well as pain due to orthodontic tooth movement [33]. The limited sample size, lack of evaluation of groups with different arch length deviations, use of different types of attachment, and various biomechanics of both treatment methods are among this study’s main limitations. Therefore, we recommend conducting new research with groups with a different arch length discrepancy using various attachment types and performing biomechanically similar treatment methods in wider populations.

## Conclusion

We found no significant difference between the two groups in terms of anxiety levels, and pain was more intense with conventional fixed orthodontic treatment at the beginning of treatment than with clear-aligner treatment. Furthermore, the OHIP-14 survey results showed that individuals with clear aligners had a higher quality of life than those who underwent conventional fixed orthodontic treatment at the beginning of treatment.

## Data Availability

The datasets used and/or analysed during the current study available from the corresponding author on reasonable request.
